# The new scope of virus taxonomy: partitioning the virosphere into 15 hierarchical ranks

**DOI:** 10.1038/s41564-020-0709-x

**Published:** 2020-04-27

**Authors:** International Committee on Taxonomy of Viruses Executive Committee, Alexander E. Gorbalenya, Mart Krupovic, Arcady Mushegian, Andrew M. Kropinski, Stuart G. Siddell, Arvind Varsani, Michael J. Adams, Andrew J. Davison, Bas E. Dutilh, Balázs Harrach, Robert L. Harrison, Sandra Junglen, Andrew M. Q. King, Nick J. Knowles, Elliot J. Lefkowitz, Max L. Nibert, Luisa Rubino, Sead Sabanadzovic, Hélène Sanfaçon, Peter Simmonds, Peter J. Walker, F. Murilo Zerbini, Jens H. Kuhn

**Affiliations:** 1 Departments of Biomedical Data Sciences and Microbiology, Leiden University Medical Centre, Leiden, the Netherlands; 2 Faculty of Bioengineering and Bioinformatics, Lomonosov Moscow State University, Moscow, Russia; 3 Archaeal Virology Unit, Institut Pasteur, Paris, France; 4 Division of Molecular and Cellular Biosciences, National Science Foundation, Alexandria, VA USA; 5 Departments of Food Science and Pathobiology, University of Guelph, Guelph, Ontario, Canada; 6 School of Cellular and Molecular Medicine, University of Bristol, Bristol, UK; 7 The Biodesign Center for Fundamental and Applied Microbiomics, Center for Evolution and Medicine, School of Life Sciences, Arizona State University, Tempe, AZ USA; 8 Minehead, UK; 9 MRC–University of Glasgow Centre for Virus Research, Glasgow, UK; 10 Theoretical Biology and Bioinformatics, Science for Life, Utrecht University, Utrecht, the Netherlands; 11 Institute for Veterinary Medical Research, Centre for Agricultural Research, Budapest, Hungary; 12 Invasive Insect Biocontrol and Behavior Laboratory, US Department of Agriculture–Agricultural Research Service, Beltsville, MD USA; 13 Institute of Virology, Charité – Universitätsmedizin Berlin, corporate member of Free University Berlin, Humboldt-University Berlin and Berlin Institute of Health, Berlin, Germany; 14 The Pirbright Institute, Pirbright, UK; 15 Department of Microbiology, University of Alabama at Birmingham, Birmingham, AL USA; 16 Department of Microbiology, Blavatnik Institute, Harvard Medical School, Boston, MA USA; 17 Istituto per la Protezione Sostenibile delle Piante, National Research Council of Italy, Bari, Italy; 18 Department of Biochemistry, Molecular Biology, Entomology and Plant Pathology, Mississippi State University, Mississippi State, MS USA; 19 Summerland Research and Development Centre, Agriculture and Agri-Food Canada, Summerland, British Columbia, Canada; 20 Nuffield Department of Medicine, University of Oxford, Oxford, UK; 21 School of Chemistry and Molecular Biosciences, The University of Queensland, St Lucia, Queensland Australia; 22 Departamento de Fitopatologia/BIOAGRO, Universidade Federal de Viçosa, Viçosa, Brazil; 23 Integrated Research Facility at Fort Detrick (IRF-Frederick), National Institute of Allergy and Infectious Diseases, National Institutes of Health, Frederick, MD USA

**Keywords:** Infectious diseases, Taxonomy, Virology, Microbial ecology, Classification and taxonomy

## Abstract

Virus taxonomy emerged as a discipline in the middle of the twentieth century. Traditionally, classification by virus taxonomists has been focussed on the grouping of relatively closely related viruses. However, during the past few years, the International Committee on Taxonomy of Viruses (ICTV) has recognized that the taxonomy it develops can be usefully extended to include the basal evolutionary relationships among distantly related viruses. Consequently, the ICTV has changed its Code to allow a 15-rank classification hierarchy that closely aligns with the Linnaean taxonomic system and may accommodate the entire spectrum of genetic divergence in the virosphere. The current taxonomies of three human pathogens, Ebola virus, severe acute respiratory syndrome coronavirus and herpes simplex virus 1 are used to illustrate the impact of the expanded rank structure. This new rank hierarchy of virus taxonomy will stimulate further research on virus origins and evolution, and vice versa, and could promote crosstalk with the taxonomies of cellular organisms.

## Abstract (web summary)

Here, the International Committee on Taxonomy of Viruses describe a new, expanded virus classification scheme with 15 ranks that closely aligns with the Linnaean taxonomic system and better encompasses viral diversity.

## Main

Viruses were discovered at the end of the nineteenth century as filterable agents causing infectious diseases of plants and animals^1–5^. Subsequently, their pathogenicity and ability to undergo rapid evolutionary change^6^ has sparked a large body of research, often connected to the so-called ‘microevolution’ of relatively closely related viruses^7,8^. However, over the last decade, our appreciation of the importance and distribution of viruses has expanded beyond the original parasitic–pathogen model, and now virologists recognize the role of viruses in host regulation and the maintenance of natural ecosystems^9^. Shotgun metagenomic sequencing has also revealed the presence of a vast variety of viruses in diverse environmental samples and in apparently healthy organisms from all divisions of life^10–13^.

To understand the true extent of virus genomic diversity—which may be significantly broader than that of their hosts—and the origins and forces that shape this diversity, virologists will have to systematically rationalize the more distant relationships between viruses, ideally reflecting their ‘macroevolution’, and virus taxonomy should provide an inclusive yet dynamic classification framework to reflect these relationships. In contrast to the taxonomies of cellular organisms, this new virus taxonomic framework will have to accommodate the current view that viruses have multiple origins (polyphyly) and that their diversity cannot be represented by a single virosphere-wide tree^14^.

## The traditional five-rank structure of virus taxonomy

**Fig. 1:**
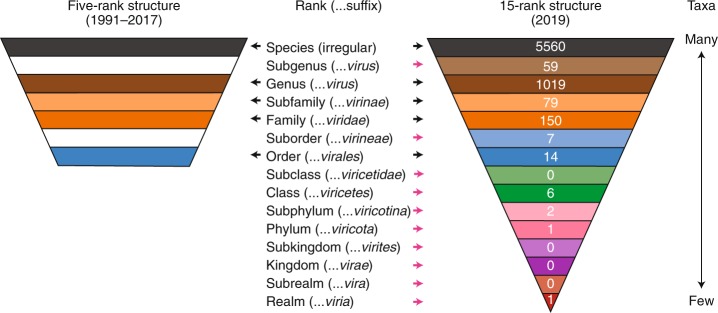
A comparison of the ICTV taxonomic rank hierarchy in 1991–2017 and 2019. Taxonomic ranks are shown in relation to the distribution pattern of taxa. The number of taxa assigned to each rank (as recorded in the current ICTV Master Species List, release 2018b, MSL34 (ref. ^47^)) are shown in white font on the 15-rank structure. When the ranks are described as a hierarchy, the species rank is often referred to as the lowest rank and the realm rank as the highest rank. However, when the ranks are used as phylogenetic terms, the realm rank can be described as basal and the species rank as apical or terminal. Both conventions are used in this Consensus Statement. Black arrows, ranks common to the five- and 15-rank structure; pink arrows, ranks introduced in the 15-rank structure.

The International Committee on Taxonomy of Viruses (ICTV) oversees the official classification of viruses and nomenclature of taxa, that is, taxonomy (Box 1)^15^. In its earliest versions, the ICTV classification of viruses into taxa formally recognized only genera and families but, over time, this classification scheme developed into a five-rank hierarchy of species, genus, subfamily (used rarely), family and order^16,17^. This five-rank structure matched a section of the Linnaean hierarchical structure used in the taxonomies of cellular organisms and remained in place until 2017 (Fig. 1, left). In addition to changes in the rank hierarchy, the recognition of virus taxa has also evolved over time, from a traditional phenotype-based characterization process to a multistage process that increasingly, but not exclusively, includes genomic properties and sequences (Box 2)^15,18^. Nowadays, formal virus classification emphasizes comparative sequence analyses of conserved genes and proteins, including gene phylogeny, gene synteny and shared gene content. Other molecular traits are also considered when appropriate^19,20^.

Box 1 The ICTV The ICTV, which was originally named the International Committee on Nomenclature of Viruses, is a voluntary, largely self-regulating and non-profit global organization. Currently, membership includes about 150 virologists representing many nationalities, with the majority of members elected or appointed for a fixed term^15^. The ICTV is a committee of the Virology Division of the International Union of Microbiological Societies (IUMS) and is governed by an Executive Committee (EC) that supervises approximately 100 specialized Study Groups. The Study Group members are chosen by each Study Group Chair, who is an ICTV member. The ICTV is responsible for developing the taxonomy, including the official classification of all viruses, viroids and satellites, regardless of their hosts or perceived importance, as well as the nomenclature of approved taxa. This taxonomy is in accordance with the Statutes, which form the normative basis of the organization, and the Code, which formalizes the rules on implementing the taxonomy. The ICTV also maintains several web-based resources that serve the virology community:

• ICTV taxonomy database (https://ictv.global/taxonomy/). The ICTV database can be accessed using an online taxonomy browser. The database is also used to generate a downloadable spreadsheet of current virus taxonomy, the Master Species List, which is published annually.

• Online ICTV Report (https://ictv.global/reports/). The online ICTV Report is a developing compendium of all current virus taxa with information describing the properties and characteristics of virus members of each taxon.

• Virus Metadata Resource (https://ictv.global/VMR/). The downloadable Virus Metadata Resource includes virus names, virus name abbreviations, isolate designations, GenBank accession numbers and host groups for exemplar viruses of each approved virus species.

Box 2 Virus taxonomy: a dynamic framework The ICTV is responsible for approval and ratification of virus taxonomic changes. As virus discovery continues, taxonomy is expected to advance and the process for soliciting changes in the taxonomy is as follows. The process begins with the submission of a taxonomic proposal (TaxoProp) to the ICTV EC. Any virologist can submit a TaxoProp addressing the creation, revision or dissolution of taxa, or nomenclature changes. Each TaxoProp is reviewed by the EC, considering the recommendations of relevant Study Groups. These Study Groups (often the authors or co-authors of TaxoProps) are tasked with the taxonomic development of specific families or other high-rank taxa as well as the development of demarcation criteria used to define taxa at different ranks. An EC-approved TaxoProp is then considered for ratification by the entire ICTV membership (see Box 1). Irrespective of whether a TaxoProp addresses virus taxonomy at the lower or higher ranks, taxonomic changes have to be substantiated. Importantly, each TaxoProp is publicly posted for comment and all comments are taken into consideration at all stages of TaxoProp evaluation. Virus taxonomy is not set in stone, and virologists may challenge any taxon or its rank assignment with new data or concepts using persuasive scientific arguments.

## Classifications outside of the ICTV taxonomic remit

Until recently, the evolutionary relationships between viruses of different families or orders were considered by the ICTV, and by many in the virology community, as being too distant to be resolved in a credible classification. Thus, there was little impetus to extend the taxonomy rank structure. The result was a taxonomy that, in profound contrast to its cellular counterparts, included many disjointed taxa, the number of which increases with the accelerating discovery of novel viruses (exceeding 100 families in 2018). However, classification efforts continued outside of the official taxonomic framework and, over the last few decades, several informal groupings such as ‘supergroups’ or ‘superfamilies’ were proposed for subsets of RNA viruses^21–23^ and DNA viruses^24–28^. These groupings relate to otherwise seemingly disparate viruses belonging to different families and have a variety of different hosts, genome types and organizations, and replication mechanisms. Importantly, these groupings have relied on distant relationships often associated with structure–function hypotheses (for example, an essential virus protein involved in virus replication or virion morphogenesis), which were then validated in subsequent experimental studies^29–31^; these provided independent support for the inferred classifications.

Also, before these developments took place, Baltimore had introduced a non-hierarchical classification of viruses which groups viruses into just seven (originally six) classes according to their genome type (double-stranded DNA, single-stranded DNA, double-stranded RNA, positive-sense RNA, negative-sense RNA, reverse-transcribing RNA and reverse-transcribing DNA) and its relation to the synthesis of mRNA^32,33^. Because of its conceptual clarity and functional foundation, this classification system is still widely used. It complements virus taxonomy by grouping viruses into meaningful classes at a different scale of virus divergence, albeit without attempting to evaluate their evolutionary relationships.

## The new Linnaean-like ranking hierarchy of virus taxonomy

In 2016, the urgency, timeliness and logistics of introducing additional ranks to the virus taxonomy hierarchy were discussed at length by the ICTV Executive Committee (EC). The discussion addressed how best to mirror the complete Linnaean taxonomy system (based on a nest of seven principal or primary ranks: species, genus, family, order, class, phylum and kingdom), how to allow for the hierarchical clustering of virus taxa in higher ranks such as orders, and whether the Baltimore classes might be adopted as taxa, perhaps at the basal ranks of the taxonomy^34^. Figuratively speaking, a taxonomic hierarchy was sought that could accommodate a virosphere-wide tree (or trees) from the roots to the tips of the branches. Because of its potential impact on the practice of virus taxonomy, the EC created a Working Group to consider the matter in more detail. An account of the process undertaken by the Working Group to propose a new taxonomy is outlined in Box 3.

The Working Group concluded that an extended, formal virus classification hierarchy should provide 15 ranks, including eight principal (or primary) ranks and seven derivative (or secondary) ranks (Fig. 1). The eight principal ranks include four that were already in use (order, family, genus and species) and four that are new: realm, kingdom, phylum and class, which are all above the order rank. The class rank in this series is not to be confused with the ‘classes’ described by Baltimore, or the typological attributes of a taxonomic rank^35^. These new principal ranks cover the entire scale of virus divergence to include the deepest virus relationships at the basal rank of realm. The large scale of virus divergence encompassed by the 15 ranks is exemplified by the newly created *Riboviria* taxon (a realm) that currently includes all RNA viruses encoding an RNA-directed RNA polymerase, including viruses of three Baltimore classes (III, IV and V)^36^.

The seven secondary ranks include the previously used subfamily rank and six new ranks that are derivatives of most of the remaining principal ranks. The exception is the species rank, which is currently not associated with a secondary rank, as no consensus on the definition of ‘subspecies’ could be reached. This new rank hierarchy and the associated nomenclature (Fig. 1, right), including defined suffixes for taxa, follow those used in the Linnaean system with a single exception. The basal rank is called ‘realm’ in virus taxonomy, rather than ‘domain’ (as in other taxonomies), reflecting a complex interrelation between virus taxonomy and its counterparts for cellular organisms.

The new rank hierarchy and its normative basis, in the form of changes to the ICTV Code, were approved by the ICTV EC and subsequently ratified by the ICTV in two votes in 2018 and 2019 (refs. ^37,38^). These changes provide the virology community with the opportunity to submit taxonomic proposals that fill the new principal and secondary ranks with defined taxa.

Box 3 Revision of the rank structure In 2016, the ICTV EC formed a Working Group to drive the modernization of the rank structure of virus taxonomy. This group produced a Consultation Report and then an official TaxoProp, which became available at the ICTV website for public discussion^48^. The Working Group considered the number of ranks that should be allowed by the International Code of Virus Classification and Nomenclature (ICVCN; ‘the Code’) under three main premises: (1) virus discovery will continue at an accelerating pace, which will help to establish links between distantly related viruses; (2) virus taxonomy will continue to develop to reflect the evolutionary relationships of viruses; and (3) advances in comparative genomics will provide the necessary tools for defining natural groups of viruses over the full scale of virus divergence. The deliberations also included a ‘thought exercise’ that used the informal hierarchical groupings of diverse RNA virus subsets (starting from poliovirus and other picornaviruses and concluding with all RNA viruses) to define the number of ranks that may be needed in a fully inclusive virus classification. The group recommended an extended rank structure of virus taxonomy that was subsequently approved by the ICTV. Virologists active in comparative genomics now have the opportunity to test their discoveries and theories on all aspects of virus classification through the ICTV-mediated process (see refs. ^49,50^) and its engagement with the wider community of virologists.

## Application and impact of the extended virus taxonomy

**Fig. 2:**
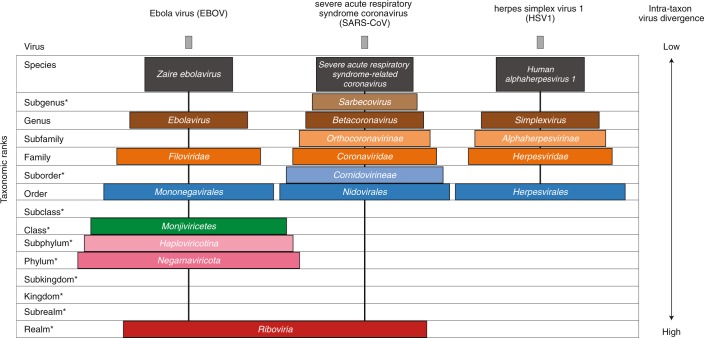
Classification of EBOV, SARS-CoV and herpes simplex virus 1 in the 15-rank taxonomic hierarchy. Intra-cluster virus divergence, which increases from the virus to the species rank to the realm rank, is represented by the increasing width of the respective rectangles, which are not drawn to scale. EBOV is most closely related to, but distinct from, Bombali, Bundibugyo, Reston, Sudan and Taï Forest viruses, which belong to separate species included in the *Ebolavirus* genus. SARS-CoV is one of several closely related coronaviruses isolated from humans and animals, such as palm civets and bats, and are included in the species *Severe acute respiratory syndrome-related coronavirus*. Herpes simplex virus 1 is one of two human herpesviruses belonging to different species in the *Simplexvirus* genus. Ranks that were introduced with the extended rank structure are indicated by an asterisk.

When developing taxonomy, virologists are only obliged to assign a (new) virus to taxa at genus and species ranks. Other ranks may be used optionally when the scientific justification is sufficient. The new 15-rank taxonomic structure applies to all viruses, although none have been assigned to all ranks thus far. To illustrate the application of some of the newly established ranks, Fig. 2 and Table 1 show the full current classification and taxa demarcation criteria of two viruses with RNA genomes, Ebola virus (EBOV) and severe acute respiratory syndrome coronavirus (SARS-CoV). Both viruses are well-known human pathogens and members of the species *Zaire ebolavirus* and *Severe acute respiratory syndrome-related coronavirus*, respectively. Although both of these viruses infect humans, they differ taxonomically in the ranks that are populated and the demarcation criteria that define the taxa. Only at the basal, realm rank are the two viruses included in the same taxon, *Riboviria*. Figure 2 also shows the corresponding information for another human pathogen, herpes simplex virus 1, which has a double-stranded DNA genome and is currently assigned to taxa of five ‘traditional’ ranks.

**Table 1:** Classification of EBOV and SARS-CoV in the 15-rank taxonomic hierarchy

	EBOV		SARS-CoV	
Rank	Taxon	Demarcation criteria	Taxon	Demarcation criteria
Species	*Zaire ebolavirus*^a^	The species of the genus that includes the human pathogen EBOV.	*Severe acute respiratory syndrome-related coronavirus*^b^	The species of the subgenus, including the human pathogen SARS-CoV, that have diverged by less than 7.6% in the 3 CLpro, NiRAN, RdRp, ZBD and HEL1 domains of the replicase protein.
Subgenus	Unassigned		*Sarbecovirus*^b^	Members of the genus that have diverged by less than 14.7% in the 3CLpro, NiRAN, RdRp, ZBD and HEL1 domains of the replicase protein.
Genus	*Ebolavirus*^a^	Members of the family that use co-transcriptional editing to express several proteins from the G (GP) ORF and encode all other proteins from monocistronic genes.	*Betacoronavirus*^b^	Members of the subfamily that have diverged by less than 36.0% in the 3CLpro, NiRAN, RdRp, ZBD and HEL1 domains of the replicase protein.
Subfamily	Unassigned		*Orthocoronavirinae*^b^	Members of the family that have diverged by less than 51.9% in the 3CLpro, NiRAN, RdRp, ZBD and HEL1 domains of the replicase protein.
Family	*Filoviridae*	Members of the order that infect vertebrates, produce filamentous virions and encode two proteins (VP30 and VP24) that do not have homologues in other order members.	*Coronaviridae*^b^	Members of the suborder that have diverged by less than 68.3% in the 3CLpro, NiRAN, RdRp, ZBD and HEL1 domains of the replicase protein.
Suborder	Unassigned		*Cornidovirineae*^b^	Members of the order that have diverged by less than 73.4% in the 3CLpro, NiRAN, RdRp, ZBD and HEL1 domains of the replicase protein.
Order	*Mononegavirales*	Members of the class that have a common, linear ORF core set (3’-N-P-M-G-L-5’).	*Nidovirales*	Members of the realm that share syntheny of 3CLpro, NiRAN, RdRp, ZBD and HEL1 domains of the replicase protein.
Subclass	Unassigned		Unassigned	
Class	*Monjiviricetes*	As the subphylum currently includes only a single class, all members of the subphylum *Haploviricotina* are members of the class *Monjiviricetes*.	Unassigned	
Subphylum	*Haploviricotina*	Members of the phylum with primary, non-segmented genomes encoding capping enzymes.	Unassigned	
Phylum	*Negarnaviricota*	Members of the realm with negative-sense RNA genomes.	Unassigned	
Subkingdom	Unassigned		Unassigned	
Kingdom	Unassigned		Unassigned	
Subrealm	Unassigned		Unassigned	
Realm	*Riboviria*	Viruses with an RNA genome encoding an RNA-directed RNA polymerase.	*Riboviria*	Viruses with an RNA genome encoding an RNA-directed RNA polymerase.

^a^Pairwise sequence comparison^42^ using coding-complete filovirus genomes is the primary tool for filovirus species and genus demarcation. Genomic sequences of ebolaviruses of different species differ from each other by ≥23%. Genomic sequences of filoviruses of different genera differ from each other by ≥55%. Genomic features, such as number and location of gene overlaps, ebolavirus host and geographic distribution, and ebolavirus pathogenicity for different organisms, are also considered for species assignment, while genomic features, such as number and location of gene overlaps, number of open reading frames (ORFs) and/or genes, filovirus host and geographic distribution, and filovirus pathogenicity for different organisms, are also taken into account for genus assignment. Phylogenetic relationships across the genus have been established from maximum likelihood trees generated using coding-complete or complete genome sequences or by analysing filovirus large protein amino acid sequences^43^.

^b^Nidovirus taxa demarcation and rank assignment were defined by DivErsity pArtitioning by hieRarchical Clustering (DEmARC) analysis, a distance-based method with improved predictive power^44^ and inferred biological relevance^45^. The analysis sought minima of clustering cost in the distribution of pairwise distances derived from a maximum likelihood tree of the concatenated 3CLPro, NiRAN, RdRP, ZBD and HEL domains of the replicase polyprotein in all available sequences of viruses of the suborder *Cornidovirineae*. These minimum numbers were converted into percentages of amino acid identity that serve as a proxy for the selected minima. For the demarcation of the suborders, the order-wide analysis was performed. Members of the species, subgenus, genus, subfamily and family taxa form phylogenetically compact lineages in the *Coronaviridae* tree. Members of the suborder taxa form phylogenetically compact lineages in the *Nidovirales* tree (see ref. ^46^). 3CLpro, 3C-like protease; NiRAN, nidovirus RdRp-associated nucleotidyltransferase; RdRp, RNA-directed RNA polymerase; ZBD, Zn-binding domain covalently linked to HEL1; HEL1, helicase of superfamily 1.

A surge of activity to populate the existing and additional ranks of virus taxonomy can now be expected and is to be encouraged. This will include the consideration of numerous supergroups and superfamilies that have remained outside of virus taxonomy to date. At the time of writing (January 2020), the ranks included highly different numbers of taxa (Fig. 1). As expected, the species rank, with the lowest degree of intra-taxon virus divergence, is the most heavily populated, and ranks with higher degrees of intra-taxon divergence are the least populated. This highly uneven distribution of the number of taxa assigned at different ranks is due not only to the hierarchical relationship of the ranks, but also to the very recent introduction of the more basal ranks, and the lack of requirement to fill these ranks. The uneven distribution also reflects variations in the sampling of different virus lineages in diverse hosts as well as differences in the approaches adopted for the recognition of taxa in particular species by different ICTV Study Groups.

These variations could persist, although the currently observed differences in taxon density may be partially alleviated when researchers define the more distant taxonomic relationships among viruses and improve their resolution by involving traditional and new evolutionary methodologies, such as network analyses^28^. For example, increasing evidence supports an ancestral relationship of some viruses of the order *Caudovirales* (a group of bacterial viruses with double-stranded DNA genomes) and viruses of the order *Herpesvirales* (a group of animal viruses with double-stranded DNA genomes) through a shared virion morphogenesis module. This module includes the HK97-type major capsid protein, portal protein, capsid maturation protease and the genome-packaging terminase complex^25,39–41^, and appears to reflect monophyletic relationships that may warrant taxonomic recognition.

As a result of the change to the number and scope of ranks, virus taxonomy is now, for the first time, able to accommodate taxa at any level of virus divergence between the very narrow (species) and the extremely wide (realms). How these ranks are used will depend on the research community, including the ICTV. We stress that the validity of any established taxa, or those created in the future, depends on the strength of scientific evidence to support the demarcation and ranking of taxa, which is considered on a case-to-case basis.

The codified availability of a greater number of ranks in a formal virus classification that emulates a Linnaean framework may also facilitate the comparison, and possibly improve the compatibility of virus taxonomy with the taxonomies of cellular organisms. Although the switching of hosts by viruses may be a complicating factor, the availability of fossils and a defined evolutionary timescale for some virus hosts should benefit virus taxonomy. Such information will be essential for taxa demarcation and rank definition in the future, notwithstanding that all taxonomies depend on the accuracy of evolutionary reconstructions, which are most challenging for distant relationships that reflect numerous changes, including those resulting from horizontal (lateral) gene transfer.

We expect that the described changes to the hierarchical rank structure will create a new impetus for the exploration of virus macroevolution and a framework for its application to taxonomy. The changes will also stimulate research on the defining characteristics of monophyletic virus lineages and the recognition of historical events that played a decisive role in their origins and evolution. These events may be comparable to major transitions in the evolution of cellular life forms, such as the origins of eukaryotes or plants. This information could be used to define taxa and ranks, and the improved interaction with evolutionary research will facilitate the main mission of virus taxonomy, which is to serve the virology community and the public at large in a comprehensive, scientific manner.
